# Retinal microvascular changes are related to indicators of glymphatic dysfunction and small vessel disease in frontotemporal dementia

**DOI:** 10.1002/alz.71844

**Published:** 2026-09-17

**Authors:** William Robert Kwapong, Yufei Chen, Xu Chen, Jiahui Hou, Hua Lu, Yuxuan Xu, Qianqian He, Yuan Gao, Ruoyu Jiao, Liyong Wu, Xuxiang Zhang, Min Chu

**Affiliations:** ^1^ Department of Neurology, Xuanwu Hospital Capital Medical University Beijing China; ^2^ Department of Ophthalmology, Xuanwu Hospital Capital Medical University Beijing China; ^3^ Department of Geriatrics National Clinical Research Center for Geriatric Diseases Beijing China

**Keywords:** cognitive decline, diffusion tensor imaging, frontotemporal dementia, glymphatic function, retinal microvasculature

## Abstract

**INTRODUCTION:**

Retinal vascular alterations have been associated with cerebral small vessel disease (SVD) in frontotemporal dementia (FTD), but their relationship with magnetic resonance imaging (MRI)‐derived glymphatic‐related diffusion markers remains unclear. We investigated retinal vascular fractal dimension (FD) in relation to SVD, glymphatic‐related diffusion metrics, and clinical measures.

**METHODS:**

Thirty‐four patients with clinically diagnosed FTD (82.4% behavioral variant FTD) and 34 cognitively unimpaired controls underwent color fundus photography and multimodal MRI. Generalized estimating equations, multivariable regression, and partial Spearman correlations were performed.

**RESULTS:**

FTD showed sparser retinal microvasculature, lower diffusion tensor imaging analysis along the perivascular space (DTI‐ALPS), and periventricular diffusivity, and higher SVD burden (all *p* < 0.05). Sparser retinal microvasculature was associated with reduced DTI‐ALPS, lower periventricular diffusivity, greater SVD burden, poorer cognition, and greater disease severity (all *p* < 0.05).

**DISCUSSION:**

Retinal microvascular alterations are related to SVD and glymphatic dysfunction in FTD.

## Highlights

- Retinal vascular changes were associated with impaired glymphatic function, as assessed by diffusion tensor imaging along the perivascular space (DTI‐ALPS) and periventricular diffusivity (PVeD).
- Increased cerebral small vessel disease (SVD) burden was found in patients with frontotemporal dementia (FTD), correlating with retinal and glymphatic abnormalities.
- Retinal microvascular changes were significantly linked to cognitive performance and disease severity in FTD.
- These findings support the potential of retinal imaging as a non‐invasive indicator of neurovascular and glymphatic dysfunction in FTD.

## 1 BACKGROUND

Frontotemporal dementia (FTD) is a heterogeneous group of neurodegenerative disorders characterized by progressive deterioration in behavior, executive function, and/or language due to selective degeneration of the frontal and temporal lobes.^1^, ^2^, ^3^ Despite considerable advances in understanding the molecular and genetic basis of FTD, the mechanisms linking neurodegeneration with cerebrovascular dysfunction remain incompletely understood. Increasing evidence suggests that vascular dysfunction may contribute to disease progression by impairing cerebral perfusion, disrupting neurovascular integrity, and altering interstitial fluid clearance pathways. ^2^, ^3^, ^4^, ^5^, ^6^

The retina shares embryological origin and microvascular characteristics with the brain and has been proposed as a readily accessible window into cerebral microvasculature.^7^, ^8^ Quantitative retinal vascular imaging has emerged as a promising biomarker for several neurological disorders including Alzheimer's disease (AD), cerebral small vessel disease (SVD), and stroke.^9^, ^10^, ^11^, ^12^, ^13^, ^14^, ^15^, ^16^ Among retinal vascular measures, fractal dimension (FD) reflects the geometric complexity and branching architecture of the retinal vascular network and has been associated with cerebral microvascular abnormalities and cognitive impairment.^10^, ^14^, ^17^, ^18^

Recently, we demonstrated that retinal microvascular alterations measured using optical coherence tomography angiography (OCTA) were associated with magnetic resonance imaging (MRI)‐derived SVD burden in patients with FTD, supporting the concept that retinal microvascular abnormalities reflect microvascular pathology ^3^. However, SVD represents only one component of the vascular component of the vascular alterations observed in neurodegenerative diseases. Emerging evidence indicates that impaired glymphatic function, which facilitates perivascular clearance of metabolic waste and maintenance of brain homeostasis, may also contribute to FTD pathophysiology.^2^, ^4^, ^6^ Whether retinal vascular alterations are related not only to structural markers of SVD but also to imaging biomarkers reflecting glymphatic‐related fluid transport remains unknown.

Diffusion tensor imaging–based approaches, including diffusion tensor imaging analysis along the perivascular space (DTI‐ALPS), provide a non‐invasive method for estimating glymphatic function by quantifying water diffusivity along perivascular pathways.^19^ More recently, periventricular diffusivity (PVeD) has been proposed as a complementary diffusion‐derived metric reflecting interstitial fluid dynamics within periventricular regions associated with perivenous drainage pathways.^20^ Integrating these complementary MRI measures with retinal vascular imaging offers an opportunity to investigate whether retinal vascular architecture reflects broader neurovascular and fluid clearance abnormalities beyond conventional SVD.

Therefore, the aim of the present study was to investigate whether retinal vascular FD is associated with MRI‐derived markers of glymphatic‐related diffusion and cerebral SVD in a clinically diagnosed, predominantly behavioral variant FTD cohort, and to determine whether these multimodal imaging measures relate to cognitive performance and disease severity.

**RESEARCH IN CONTEXT**

1. **Systematic review**: We reviewed the literature on retinal microvascular alterations, glymphatic system dysfunction, and cerebral small vessel disease (SVD) in neurodegenerative disorders using databases including PubMed and Web of Science. Prior studies have demonstrated that retinal vascular metrics, particularly fractal dimension, are altered in Alzheimer's disease and are associated with cerebral SVD markers. Emerging evidence also supports the role of glymphatic dysfunction, assessed using diffusion tensor imaging analysis along the perivascular space (DTI‐ALPS), in Alzheimer's disease and aging. However, studies investigating these mechanisms in frontotemporal dementia (FTD) are limited, and the relationship between retinal microvasculature and glymphatic‐related imaging markers has not been well characterized in this population.
2. **Interpretation**: This study demonstrates that patients with FTD exhibit reduced retinal microvascular complexity, impaired glymphatic‐related diffusion metrics, and increased SVD burden. Importantly, retinal microvascular alterations were significantly associated with both glymphatic dysfunction and SVD, as well as with cognitive performance and disease severity. These findings suggest that retinal microvasculature may reflect underlying neurovascular and fluid clearance abnormalities in FTD and provide evidence linking vascular and glymphatic mechanisms in this disorder.
3. **Future directions**: Longitudinal studies are needed to determine the temporal relationships among retinal microvascular changes, glymphatic dysfunction, and disease progression in FTD. Larger, multicenter cohorts and integration with molecular biomarkers will be important to validate retinal imaging as a non‐invasive biomarker. Further mechanistic studies are also warranted to elucidate the interplay between vascular dysfunction and impaired fluid clearance pathways in neurodegeneration.

## 2 METHODS

This study was conducted as part of an ongoing prospective multimodal imaging study of FTD at the Department of Neurology, Xuanwu Hospital, Capital Medical University. Between February 2025 and January 2026, consecutively recruited participants underwent standardized clinical evaluation, color fundus photography (CFP), structural and diffusion MRI, and neuropsychological assessment.

The present investigation extends our previous work examining retinal microvascular alterations and SVD in FTD by addressing a distinct research question. Specifically, whereas our earlier study evaluated retinal capillary perfusion using OCTA in relation to MRI‐derived SVD burden, the current study quantified retinal vascular FD from CFP and investigated its relationship with MRI‐derived markers of glymphatic‐related diffusion (DTI‐ALPS and PVeD) together with cumulative SVD burden. There is no partial overlap between the two studies because participants were recruited at different times from an ongoing observational FTD cohort. The retinal imaging modality, neuroimaging measures, primary hypotheses, and statistical analyses are substantially different.

Demographic information, including age, sex, education, hypertension, diabetes mellitus, and other vascular risk factors, was recorded at study enrollment.

### 2.1 Study participants

Patients with FTD were consecutively recruited from the Neurology Department of Xuanwu Hospital. All diagnoses were established during multidisciplinary consensus meetings involving experienced behavioral neurologists according to internationally accepted diagnostic criteria.

Behavioral variant FTD (bvFTD) was diagnosed according to the revised International Consensus Criteria,^21^ while the non‐fluent variant primary progressive aphasia (nfvPPA) and semantic variant primary progressive aphasia (svPPA) fulfilled a diagnostic criteria.^22^

Because neuropathological confirmation was unavailable, the present cohort represents clinically diagnosed FTD. Patients underwent comprehensive neurological examination, neuropsychological assessment, and structural MRI to exclude alternative neurodegenerative disorders. Individuals with clinical features consistent with AD, dementia with Lewy bodies (DLB), vascular dementia, or other neurological disorders were excluded. Although amyloid positron emission tomography or cerebrospinal fluid biomarkers were not routinely available, extensive clinical evaluation was performed to minimize diagnostic uncertainty.

Thirty‐four patients met the eligibility criteria, comprising 28 patients with bvFTD, 1 patient with nfvPPA, and 5 patients with svPPA. Accordingly, the study cohort predominantly represents bvFTD.

Cognitively unimpaired (CU) controls were recruited from community volunteers undergoing routine health examinations. Controls demonstrated normal cognitive performance and no clinically significant abnormalities on neurological examination or MRI.

The same exclusion criteria were applied to both patients and controls unless otherwise specified. Participants were excluded if they had: (1) AD, DLB, vascular dementia, or other neurological disorders (controls); (2) previous stroke or major psychiatric illness; (3) uncontrolled hypertension or uncontrolled diabetes mellitus; (4) ocular diseases known to affect retinal vascular morphology, including diabetic retinopathy, hypertensive retinopathy, age‐related macular degeneration, glaucoma, optic neuropathy, optic neuritis, retinal vascular occlusion, retinal hemorrhage, severe cataract, or other media opacities affecting retinal image quality; (5) active malignancy; (6) history of substance abuse.

Participants with medically controlled hypertension or diabetes mellitus were eligible for inclusion provided that retinal complications were absent.

### 2.2 Neuroimaging with MRI

A standardized protocol was conducted using a 3.0 T MRI system (SIGNA MR, GE Healthcare) in all participants, including 3D T1‐weighted image, axial T2‐weighted image, and axial T2 fluid‐attenuated inversion recovery (FLAIR) image. For the present study, the MRI data were collected using a 19‐channel head and neck union coil provided by the vendor. The protocol included both a 3D T1‐weighted sagittal scan and diffusion tensor imaging (DTI) scans. The acquisition parameters for the scans were as follows: (1) A 3D T1‐weighted fast field echo sequence with a repetition time (TR) of 6.9 ms, echo time (TE) of 2.98 ms, flip angle of 12°, inversion time of 450 ms, matrix size of 256 × 256, field of view (FOV) of 256 × 256 mm^2^, slice thickness of 1 mm, 192 sagittal slices with no gap, and voxel size of 1 × 1 × 1 mm^3^; (2) diffusion‐weighted spin‐echo echo‐planar imaging sequence with a *b* value of 0 s/mm^2^ and 30 directions with a *b* value of 1000 s/mm^2^, TR of 16500 ms, TE of 74.6 ms, matrix size of 112 × 112, FOV of 256 × 256 mm^2^, slice thickness of 2 mm, 70 axial slices with no gap, and a single excitation.

#### 2.2.1 Visual rating of SVD burden

SVD markers on MRI—including lacunes, cerebral microbleeds, white matter hyperintensities (WMHs), and perivascular spaces (PVSs)—were assessed in accordance with the Standards for Reporting Vascular Changes on Neuroimaging (STRIVE) consensus guidelines.^23^ Lacunes were defined as rounded or ovoid cavities located in subcortical regions, measuring between 3 and 15 mm in diameter, displaying cerebrospinal fluid–like signal intensity on T2‐weighted and FLAIR sequences. These lesions typically showed a hyperintense rim on FLAIR imaging and lacked diffusion restriction on diffusion‐weighted imaging. Cerebral microbleeds were identified on susceptibility‐weighted imaging as homogeneous, rounded hypointense lesions with diameters ranging from 2 to 10 mm. WMHs were assessed on FLAIR images using the Fazekas grading system, in which severity was scored separately for deep and periventricular white matter on a scale from 0 to 3; the combined score represented the overall WMH burden. PVSs were characterized as small (generally < 3 mm), round or linear hyperintense structures on T2‐weighted images located in the basal ganglia or centrum semiovale. These were graded on a validated semiquantitative scale ranging from 0 to 4. Finally, an overall cerebral SVD burden score was calculated on an ordinal scale from 0 to 4 to represent the cumulative presence of these imaging markers, following previously established criteria.^24^

#### 2.2.2 DTI processing

Diffusion‐weighted images (DWIs) were processed using DSI Studio (Chen, version released March 18, 2023; https://dsi‐studio.labsolver.org/). When available, images acquired with reversed phase‐encoding directions were first corrected for susceptibility‐induced distortions using FSL's TOPUP. Subsequently, eddy current–related distortions and subject head motion were corrected with FSL's EDDY. All FSL‐based corrections (https://fsl.fmrib.ox.ac.uk/fsl/) were performed through the integrated preprocessing pipeline implemented in DSI Studio. After preprocessing, the corrected DWIs were reconstructed using q‐space diffeomorphic reconstruction (QSDR),^25^ an extension of generalized q‐sampling imaging that enables non‐linear spatial normalization to the ICBM152 adult template and reconstruction of diffusion data in Montreal Neurological Institute (MNI) space.^26^ To estimate diffusivity associated with fast diffusion components, only DWIs with *b* values ≤ 1000 s/mm^2^ were included in the diffusion tensor model. The orientation of the b‐table was validated by comparing reconstructed fiber directions to those from a population‐averaged brain template.^27^ A diffusion sampling length ratio of 1.25 was applied, and the final QSDR output had an isotropic voxel size of 2 mm. From the tensor model, color‐encoded fractional anisotropy (FA), mean diffusivity (MD), and directional diffusivity maps (Dxx, Dyy, and Dzz) were derived. DWIs with low signal‐to‐noise ratio, poor spatial correspondence with adjacent volumes, unsuccessful distortion correction, or visible artifacts were excluded. In addition, reconstructed datasets showing low spatial agreement with the template were removed from further analysis.

#### 2.2.3 Calculation of DTI‐ALPS

Regions of interest (ROIs) for the DTI‐ALPS analysis were defined using a group‐averaged template constructed in MNI space from reconstructed DWIs obtained from the Lifespan Human Connectome Project in Aging,^19^, ^28^ accessed via the DSI Studio data‐sharing platform (https://brain.labsolver.org/hcp_a.html).^29^ On the template's color‐coded FA maps, projection and association fiber pathways were visually identified. Spherical ROIs with a radius of 3 mm were then positioned bilaterally within these fiber regions at the level of the body of the lateral ventricles.

In total, four ROIs were applied to each subject's axis‐specific diffusivity maps, and diffusivity measures (Dxx, Dyy, and Dzz) were extracted from these regions for subsequent DTI‐ALPS computation. The DTI‐ALPS metric was calculated as the ratio of the MD along the *x* axis within projection fibers (Dxx‐proj) and association fibers (Dxx‐assoc) to the MD along the *y* axis in projection fibers (Dyy‐proj) and along the *z* axis in association fibers (Dzz‐assoc). DTI‐ALPS indices were derived separately for the left and right hemispheres, as well as averaged across hemispheres, to characterize diffusion properties associated with glymphatic system function.^28^

#### 2.2.4 Estimation of PVeD

**FIGURE 1:**
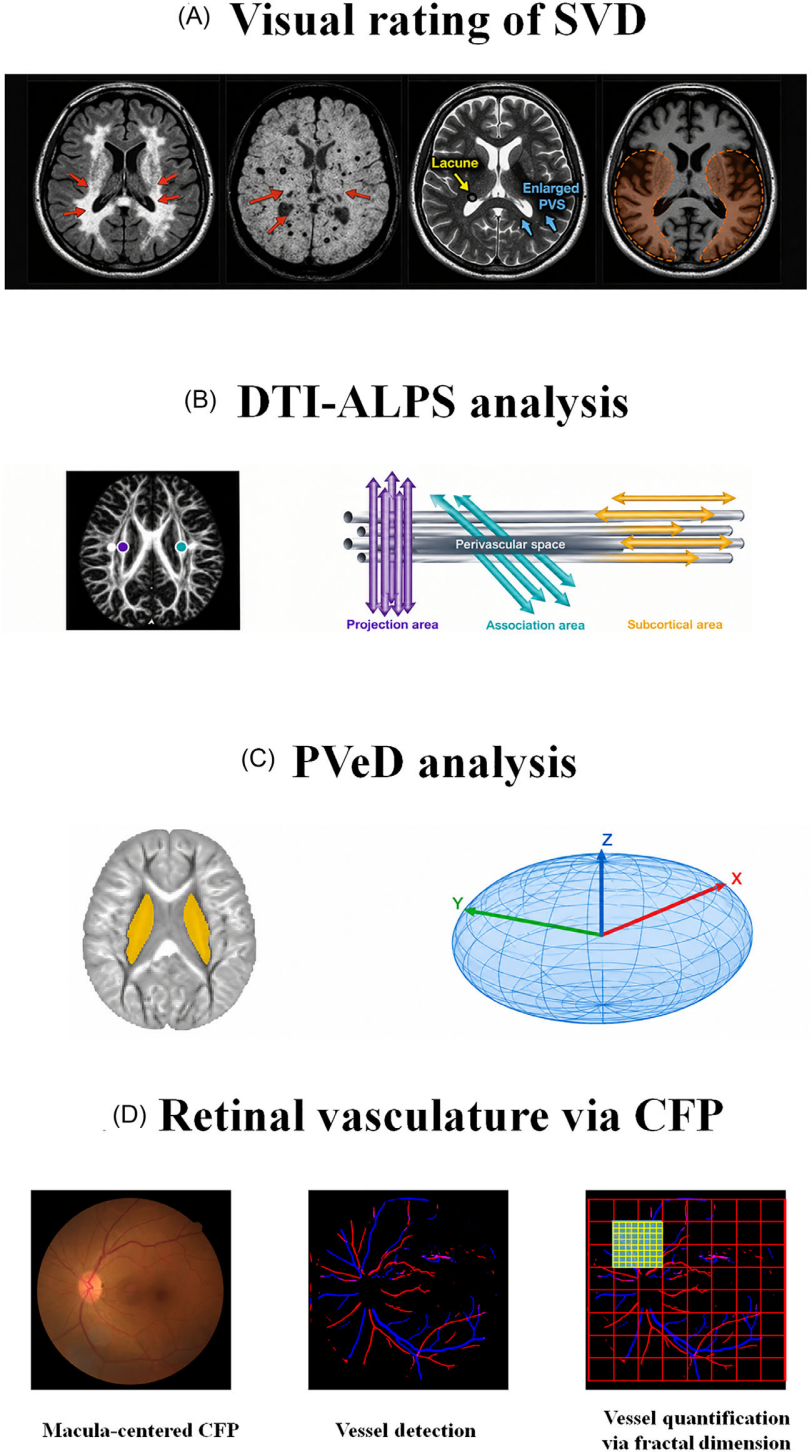
Multimodal imaging of cerebral small vessel disease, glymphatic function, and retinal microvasculature. A, Visual rating of cerebral small vessel disease. B, The diffusion tensor image analysis along the perivascular space (DTI‐ALPS) method. The schematic illustrates the measurement of water diffusivity along the direction of the perivascular space (*x* axis) within projection and association fibers to estimate glymphatic clearance efficiency. To account for anatomical variability, colors have been assigned to specific tissue compartments: green (projection fibers), yellow (association fibers), pink (focal regions), and purple (periventricular cerebrospinal fluid). C, Periventricular diffusivity (PVeD) quantification is performed by measuring mean diffusivity within periventricular regions (blue). D, Retinal vasculature via CFP: retinal microvascular complexity is derived from color fundus photography (CFP). Left, A macula‐centered fundus photo is processed through (middle) automated vessel detection and (right) box counting‐based quantification to calculate the retinal fractal dimension.

An automated approach to estimate transverse fluid diffusivity in the periventricular region (PVeD), which is hypothesized to capture fast diffusion processes within the perivenous space and to reflect glymphatic clearance integrity, was performed. To characterize diffusion along the left‐right–oriented deep medullary veins, we introduced a voxel‐wise metric termed the transverse tensor ratio (TTR), defined as the ratio of the *x* axis tensor component (Dxx) to the Euclidean norm of the full diffusion tensor (Dxx, Dyy, Dzz). This measure emphasizes the transverse component of water diffusion relative to orthogonal directions (Figure 1).

An automatic region‐growing procedure was implemented to delineate periventricular areas containing deep medullary veins using MD maps. White matter masks were first generated from MD images, and a predefined lateral ventricle mask was registered to individual MD maps to account for subject‐specific ventricular anatomy. This ventricle mask was then expanded along the transverse axis using an anatomically informed dilation scheme, with maximal expansion at the ventricular body and minimal expansion near the ventricular margins. The resulting periventricular area mask was intersected with the white matter mask to ensure sampling within white matter tissue only.

The median TTR value within the final periventricular mask was calculated as PVeD, representing the average transverse interstitial fluid diffusivity in the periventricular region of the left and right hemispheres. All processing was performed using SPM12 and CAT12 in MATLAB (R2023a). The implementation code for PVeD estimation is publicly available (https://github.com/ChangleChen/EstPVeD.html), and detailed mathematical formulation and validation of the region‐growing method have been reported previously.^20^

### 2.3 Retinal vascular imaging via CFP

Retinal and optic disc‐centered photographs were taken of each eye with a digital camera (CLARUS 500, Carl Zeiss Meditec AG). For all participants, macula‐centered images (2576 ×1934 pixels) from both eyes were obtained without pupil dilation. All photographs were assessed by a trained, certified grader masked to participant characteristics (X.C.). Images were considered gradable if they demonstrated adequate focus, illumination, contrast, complete visualization of the retinal vascular network within the analysis region, and absence of significant motion artifact or media opacity. Images not meeting these predefined quality criteria were excluded before quantitative analysis. Participants with retinopathy, such as age‐related macular degeneration, severe cataract, glaucoma, optic neuritis, and hemorrhages, were excluded.

In this study, a methodology using the MATLAB engine invoked from Python was used to extract retinal vascular features as previously reported.^10^, ^11^, ^14^, ^30^ FD was used in our study. FD serves as a geometric complexity metric for retinal vascular branching patterns, in which elevated values denote a more elaborate and space‐filling architecture of the microvascular network. The reliability of the algorithm used in quantifying retinal microvasculature was described in a previous study.^30^, ^31^ Additionally, a novel semi‐supervised approach was proposed by a previous report to differentiate arterioles and venules and obtain their FD (Figure 1D). If a participant presented with any of these disorders in one eye, the other eye was used; if both eyes had the aforementioned disorders, the participant was excluded from the study.

### 2.4 Statistical analyses

Descriptive statistics were used to summarize demographic and clinical characteristics across the three diagnostic groups. Group differences in continuous variables were evaluated using one‐way analysis of variance (ANOVA), while categorical variables were compared using chi‐squared tests. Group comparisons of neuropsychological and neuroimaging measures (DTI‐ALPS, PVeD, and SVD burden) were examined using analysis of covariance (ANCOVA), controlling for age, sex, and education.

A multiple linear regression model with a generalized estimating equation (GEE) was used to compare the retinal microvascular measure (FD) between CU and FTD while adjusting for risk factors. GEE was also used to compare the retinal microvascular measure (FD) between FTD with and without pathogenic gene mutation. The covariates were set as age, sex, vascular risk factors (hypertension), and education years, and the working correlation matrix was set as exchangeable, considering the correlation of the two eyes.

GEE was also used to explore the association of retinal microvasculature with neuroimaging measures (DTI‐ALPS, PVeD, and SVD burden) and core FTD characteristics, which included global cognitive performance (Mini‐Mental State Examination [MMSE] and Montreal Cognitive Assessment [MoCA]) and disease severity (global frontotemporal lobar degeneration Clinical Dementia Rating [FTLD‐CDR]). Analyses were adjusted for age, sex, hypertension, and education years. Results were reported as regression coefficients (ß) with corresponding 95% confidence intervals (CIs). Analyses were also conducted separately for the left (OS) and right (OD) eyes using general linear models to ensure the transparency of the results.

To explore the association between neuroimaging measures (DTI‐ALPS, PVeD, and SVD burden) and core FTD characteristics, a partial Spearman correlation analysis was performed while adjusting for covariates. Analyses were adjusted for age, sex, hypertension, and education years. Results were reported as regression coefficients (*r*) with corresponding 95% CIs.

We further tested the interaction between retinal vasculature and neuroimaging measures on global cognitive performance by including the cross‐product term of retinal vasculature × neuroimaging features along with the main effect terms of each variable in the models. The same covariates used in previous analyses examining the relationship between neuroimaging measures and global cognitive performance were controlled. For all models, *p* values < 0.05 were considered statistically significant. All statistical analyses were performed using MATLAB R2023a (The MathWorks Inc.) and R software (version 4.4.4).

## 3 RESULTS

### 3.1 Participant characteristics

A total of 68 participants were included, comprising 34 patients with clinically diagnosed FTD and 34 CU controls. The FTD cohort was predominantly composed of patients with bvFTD (*n* = 28, 82.4%), with 1 patient diagnosed with nfvPPA and 5 with svPPA.

Demographic and clinical characteristics are summarized in Table 1. Age, sex distribution, years of education, hypertension, and diabetes mellitus did not differ significantly between the two groups (all *p* > 0.05). As expected, patients with FTD demonstrated significantly lower MMSE and MoCA scores and higher FTLD‐CDR global scores than CU participants (all *p* < 0.001).

**TABLE 1:** Baseline information and clinical data of study participants.

	FTD	CU	*p*
Demographics			
Number	34	34	
Eyes, *n*	68	68	
Age, years	59.97 ± 7.61	60.53 ± 6.76	0.750
Males, *n* (%)	23 (67.65%)	15 (44.12%)	0.087
Duration, years	2 [2, 3]		
Education, years	12 [9, 15]	9 [9, 14]	0.854
Disease phenotype			
Behavioral variant, *n* (%)	28 (82.35%)		
Progressive non‐fluent aphasia, *n* (%)	1 (2.94%)		
Semantic dementia, *n* (%)	5 (14.71%)		
Pathogenic mutation, *n* (%)	11 (32.35%)		
Vascular risk factors			
Hypertension, *n* (%)	10 (29.41%)	10 (29.41%)	1
Diabetes mellitus, *n* (%)	6 (17.65%)	9 (26.47%)	0.559
Global cognitive measures			
MMSE	23 [19, 27]	30 [29, 30]	< 0.001
MoCA	15 [10, 20]	29 [27, 30]	< 0.001
Disease severity			
FTLD‐CDR	1 [1, 2]	0	< 0.001
^a^Retinal vasculature			
Fractal dimension	1.42 ± 0.09	1.46 ± 0.03	0.001
Arterioles	1.26 ± 0.08	1.30 ± 0.02	0.001
Venules	1.31 ± 0.07	1.35 ± 0.03	< 0.001
MRI measures			
SVD burden	1 [0, 2]	0 [0, 1]	< 0.001
DTI‐ALPS	1.35 ± 0.18	1.55 ± 0.06	< 0.001
Right	1.32 ± 0.18	1.59 ± 0.07	< 0.001
Left	1.39 ± 0.22	1.51 ± 0.06	< 0.001
PVeD	0.46 ± 0.05	0.50 ± 0.04	< 0.001
Right	0.47 ± 0.05	0.50 ± 0.04	< 0.001
Left	0.44 ± 0.05	0.51 ± 0.04	< 0.001

Abbreviations: CDR, Clinical Dementia Rating; CU, cognitive unimpaired; DTI‐ALPS, diffusion tensor image analysis along the perivascular space; FTLD, frontotemporal lobar degeneration; MMSE, Mini‐Mental State Examination; MoCA, Montreal Cognitive Assessment; MRI, magnetic resonance imaging; PVeD, periventricular diffusivity; SVD, small vessel disease.

^a^ Generalized estimating equation models were used to compare the retinal vascular metrics between FTD and CU while adjusting for age, sex, hypertension, education years, and inter‐eye dependencies.

Among the FTD cohort, 11 participants (32.4%) carried pathogenic mutations. Because of the limited number of mutation carriers, analyses involving genetic status were considered exploratory.

### 3.2 Comparison of retinal microvasculature between FTD and CU

**FIGURE 2 alz71844-fig-0002:**
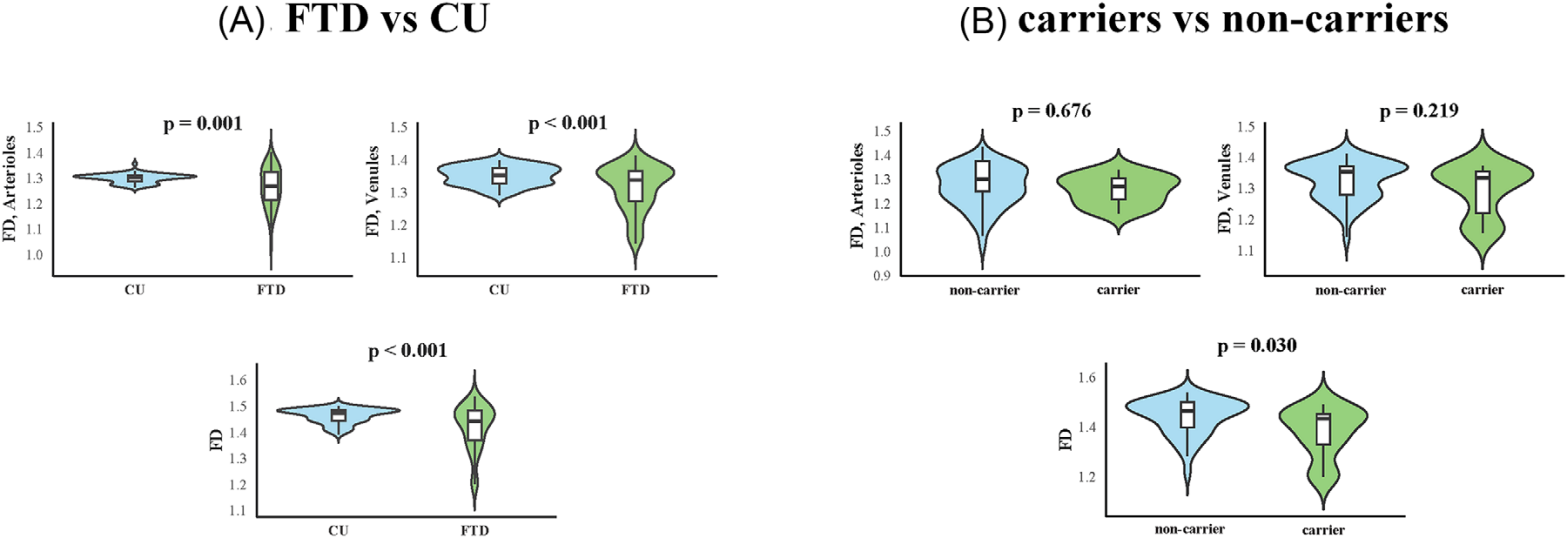
Comparison of retinal microvasculature measures. Violin and box plots illustrate the distribution of retinal microvasculature stratified by CU and FTD (A) and FTD with (carriers) and without pathogenic mutation (non‐carriers) as shown in (B). CU, cognitive unimpaired; FTD, frontotemporal dementia.

Compared to CU, FTD showed significantly sparser retinal microvasculature (1.42 ± 0.09 vs. 1.46 ± 0.03, *p* = 0.001). Correspondingly, retinal arterioles 1.26 ± 0.08 vs. 1.30 ± 0.02, *p* = 0.001) and venules 1.31 ± 0.07 vs. 1.35 ± 0.03, *p* < 0.001) were significantly sparser in FTD compared to CU. Figure 2 displays the comparison of retinal microvasculature between FTD and CU.

### 3.3 Comparison of neuroimaging measures between FTD and CU

Compared to CU participants, patients with FTD demonstrated significantly lower mean DTI‐ALPS indices and lower PVeD values (all *p* < 0.001). FTD participants also exhibited significantly greater total SVD burden than CU. These differences remained significant after adjustment for age, sex, and education (Table 1).

### 3.4 Exploratory analysis according to pathogenic mutation status

Among patients[Fig alz71844-fig-0002] with FTD, mutation carriers demonstrated significantly sparser retinal microvasculature than non‐carriers after adjustment for relevant covariates. No statistically significant differences were observed between mutation carriers and non‐carriers for DTI‐ALPS, PVeD, or total SVD burden as shown in Figure 2. Given the relatively small number of mutation carriers and the biological heterogeneity of the underlying mutations, these findings should be considered exploratory.

### 3.5 Correlation between retinal vasculature and neuroimaging measures in FTD

**FIGURE 3:**
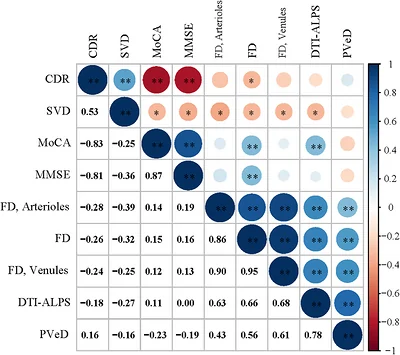
Correlation matrix of neuroimaging, retinal imaging, and clinical measures in FTD. The correlogram illustrates the Pearson correlation coefficient (*r*) and significance levels between the measures. The color scale indicates the correlation strength (blue = positive, red = negative). *p < 0.05; **p < 0.01. CDR, Clinical Dementia Rating; CU, cognitive unimpaired; DTI‐ALPS, diffusion tensor image analysis along the perivascular space; FD, fractal dimension; FTD, frontotemporal dementia; MMSE, Mini‐Mental State Examination; MoCA, Montreal Cognitive Assessment; PVeD, periventricular diffusivity; SVD, small vessel disease.

Table 2 and Figure 3 show the correlation between retinal vasculature and neuroimaging measures in FTD. We found that sparser retinal vasculature in FTD was significantly associated with lower DTI‐ALPS index (mean: ß = 0.048, 95% CI 0.018 to 0.079, *p* = 0.004; left side: ß = 0.041, 95% CI −0.019 to 0.100, *p* = 0.209; right side: ß = 0.050, 95% CI 0.010 to 0.089, *p* = 0.031) and PVeD values (mean: ß = 0.037, 95% CI 0.005 to 0.070, *p* = 0.034; left side: ß = 0.031, 95% CI −0.027 to 0.089, *p* = 0.316; right side: ß = 0.022, 95% CI −0.022 to 0.066, *p* = 0.350). We also found that sparser retinal vasculature was associated with elevated SVD burden in FTD (ß = −0.033, 95% CI −0.055 to 0.012, *p* = 0.004). Further, we found that sparser retinal arterioles were associated with lower DTI‐ALPS index (mean: ß = 0.056, 95% CI 0.024 to 0.088, *p* = 0.002) and increased SVD burden (ß = −0.037, 95% CI −0.058 to –0.015, *p* = 0.002). We also showed that sparser retinal venules were associated with lower DTI‐ALPS index (mean: ß = 0.046, 95% CI 0.021 to 0.071, *p* = 0.001), PVeD (mean: ß = 0.042, 95% CI −0.016 to 0.068, *p* = 0.004), and increased SVD burden (ß = −0.027, 95% CI −0.046 to −0.009, *p* = 0.006). After correction for multiple comparisons using the Bonferroni correction (*p* = 0.05/3 ≈ 0.017), the principal retinal–MRI associations remained statistically significant in DTI‐ALPS and SVD burden. The relationships between the left and right eyes with neuroimaging measures are shown in Table S1 in supporting information.

**TABLE 2:** Association between retinal vasculature and neuroimaging measures in FTD via generalized estimating equation models.

FD	ß (95% CI)	*p*	Arterioles	ß (95% CI)	*p*	Venules	ß (95% CI)	*p*
DTI‐ALPS	0.048 (0.018–0.079)	**0.004**	DTI‐ALPS	0.056 (0.024–0.088)	**0.002**	DTI‐ALPS	0.046 (0.021 – 0.071)	**0.001**
PVeD	0.037 (0.005–0.070)	**0.034**	PVeD	0.031 (−0.006–0.068)	0.111	PVeD	0.042 (0.016 – 0.068)	**0.004**
SVD burden	−0.033 (−0.55–−0.012)	**0.004**	SVD burden	−0.037 (−0.058–−0.015)	**0.002**	SVD burden	−0.027 (−0.046–−0.009)	**0.006**

*Note*: Data were adjusted for age, sex, hypertension, education years, and inter‐eye dependencies.

Abbreviation: CI, confidence interval; DTI‐ALPS, diffusion tensor imaging analysis along the perivascular space; FD, fractal dimension; FTD, frontotemporal dementia; PVeD, periventricular diffusivity; SVD, small vessel disease.

### 3.6 Association of retinal microvasculature and neuroimaging with global cognitive performance and disease severity in FTD

We found that sparser retinal microvasculature was associated with lower MMSE (ß = 2.120, 95% CI 0.490 to 3.750, *p* = 0.014), MoCA (ß = 1.871, 95% CI 0.175 to 3.566, *p* = 0.035), and higher FTLD‐CDR (ß = −0.413, 95% CI −0.623 to −0.203, *p* < 0.001) scores. We also found that lower DTI‐ALPS values were significantly associated with lower MoCA scores in FTD (*r* = 0.285, 95% CI 0.058 to 0.569, *p* = 0.040; Figure 3). However, no significant association was seen between PVeD values and global cognitive performance in FTD. Table 3 shows the association between neuroimaging measures and global cognitive performance and disease severity.

**TABLE 3 alz71844-tbl-0003:** Association between neuroimaging measures with global cognitive performance and disease severity in FTD via partial Spearman analysis.

MMSE	*r* (95% CI)	*p*	MoCA	*r* (95% CI)	*p*	CDR	*r* (95% CI)	*p*
DTI‐ALPS	0.150 (0.198–0.465)	0.397	DTI‐ALPS	0.285 (0.058–0.569)	**0.040**	DTI‐ALPS	−0.110 (−0.432–0.237)	0.535
PVeD	0.020 (−0.320–0.356)	0.909	PVeD	0.001 (−0.0037–0.339)	0.995	PVeD	−0.191 (−0.497–0.157)	0.280
SVD burden	−0.301 (−0.580–0.042)	0.084	SVD burden	−0.247 (−0.540–0.099)	0.158	SVD burden	0.453 (0.135–0.686)	**0.007**

*Note*: Data were adjusted for age, sex, hypertension, and education years.

Abbreviations: CDR, Clinical Dementia Rating; CI, confidence interval; DTI‐ALPS, diffusion tensor imaging analysis along the perivascular space; FTD, frontotemporal dementia; MoCA, Montreal Cognitive Assessment; PVeD, periventricular diffusivity; SVD, small vessel disease.

### 3.7 Interaction between retinal vasculature and DTI‐ALPS on FTD core characteristics

**FIGURE 4 alz71844-fig-0004:**
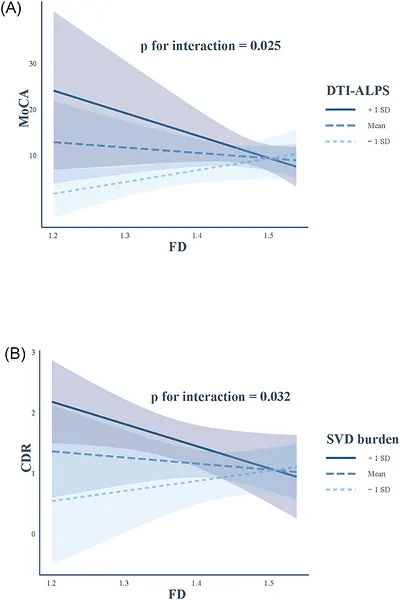
Interaction effects of retinal microvasculature and neuroimaging measures on neuropsychological measures in FTD. A, Interaction of retinal microvasculature and DTI‐ALPS on global cognitive performance (MoCA); (B) Interaction of retinal microvasculature and SVD burden on global CDR. CDR, Clinical Dementia Rating; DTI‐ALPS, diffusion tensor image analysis along the perivascular space; FD, fractal dimension; FTD, frontotemporal dementia; MoCA, Montreal Cognitive Assessment; SD, standard deviation; SVD, small vessel disease.

When the cross‐product of the retinal vasculature and DTI‐ALPS was included in the regression model with global cognitive performance as the outcome, the interaction was significant between retinal vasculature and DTI‐ALPS on MoCA scores (ß = −0.504, 95% CI −0.920 to −0.088, *p* = 0.025) as shown in Table 4. We also found a significant interaction between retinal vasculature and SVD burden (ß = −0.194, 95% CI −0.367 to −0.021, *p* = 0.032; Figure 4) on disease severity (CDR) in FTD. No significant interaction was seen between retinal vasculature with PVeD and SVD burden on cognitive performance in FTD. Because of the relatively modest sample size, these interaction analyses should be considered exploratory and interpreted cautiously.

**TABLE 4 alz71844-tbl-0004:** Interactive effect of retinal vasculature, neuroimaging measures on neuropsychological measures.

	ß (95% CI)	*p*
*MoCA*		
FD × DTI‐ALPS	−0.504 (−0.920– −0.088)	**0.025**
FD × PVeD	−0.151 (−0.381–0.079)	0.209
FD × SVD burden	0.180 (−0.008–0.369)	0.066
*MMSE*		
FD × DTI‐ALPS	−0.612 (−1.167– −0.057)	0.040
FD × PVeD	−0.198 (−0.490–0.094)	0.195
FD × SVD burden	0.135 (−0.058–0.328)	0.177
*FTLD‐CDR*		
FD × DTI‐ALPS	0.470 (0.055–0.885)	0.035
FD × PVeD	0.058 (−0.170–0.286)	0.621
FD × SVD burden	−0.194 (−0.367– −0.021)	**0.032**

*Note*: Data were adjusted for age, sex, hypertension, and education years.

Abbreviations: CDR, Clinical Dementia Rating; CI, confidence interval; FD, fractal dimension; FTLD, frontotemporal lobar degeneration; DTI‐ALPS, diffusion tensor imaging analysis along the perivascular space; MMSE, Mini‐Mental State Examination; MoCA, Montreal Cognitive Assessment; PVeD, periventricular diffusivity; SVD, small vessel disease.

## 4 DISCUSSION

In this cross‐sectional multimodal imaging study, we investigated the relationships among retinal vascular FD, MRI‐derived markers of glymphatic‐related diffusion, and SVD in a clinically diagnosed, predominantly bvFTD cohort. Three principal observations emerged. First, patients with FTD demonstrated sparser retinal microvasculature, lower DTI‐ALPS and PVeD, and greater SVD burden than CU controls. Second, sparser retinal microvasculature was associated with lower DTI‐ALPS, lower PVeD, and greater SVD burden in FTD. Third, sparser retinal microvasculature and lower DTI‐ALPS were associated with worse cognitive performance and greater disease severity. Collectively, these findings suggest that retinal vascular complexity is associated with MRI‐derived markers of cerebral vascular and fluid transport abnormalities in FTD.

Importantly, the present study extends our previous work rather than replicating it. Previously, we demonstrated that retinal capillary alterations measured using OCTA were associated with MRI‐derived cerebral SVD burden in FTD.^3^ The current study addressed a different biological question by quantifying retinal vascular architecture using CFP‐derived FD and integrating this measure with complementary MRI‐derived indices of glymphatic‐related diffusion, including DTI‐ALPS and PVeD. Whereas the previous study focused primarily on retinal capillary perfusion and conventional SVD markers, the present work investigated whether retinal vascular architecture is associated with multiple imaging markers reflecting vascular injury together with putative abnormalities in perivascular fluid transport. These complementary approaches provide a broader assessment of neurovascular alterations in FTD and support further investigation of retinal imaging as a component of multimodal[Table alz71844-tbl-0003], [Table alz71844-tbl-0004] biomarker development.

Sparser retinal microvasculature observed in the present study is consistent with growing evidence that alterations in retinal vascular architecture accompany neurodegenerative disease. Numerous studies in AD, SVD, and stroke have reported sparser retinal microvasculature, suggesting that retinal vascular remodeling may occur in parallel with cerebral microvascular abnormalities.^9^, ^10^, ^11^, ^14^, ^32^, ^33^ Because the retina and brain share common embryological origins and similar microvascular organization, retinal vascular imaging offers a practical and non‐invasive approach for studying neurovascular changes.

Another important observation was the association between retinal microvasculature and MRI‐derived glymphatic‐related diffusion metrics. DTI‐ALPS has emerged as a non‐invasive imaging surrogate reflecting water diffusivity along perivascular pathways, while PVeD has recently been proposed as a complementary marker reflecting periventricular interstitial fluid dynamics.^20^ In the present study, sparser retinal microvasculature was associated with lower values of both diffusion‐derived measures. Although the biological interpretation of these imaging biomarkers continues to evolve, our findings suggest that retinal vascular architecture and MRI‐derived markers of perivascular fluid transport may capture related aspects of neurovascular dysfunction in FTD. These observations expand previous retinal imaging studies, which have largely focused on vascular pathology alone, by incorporating imaging markers associated with brain fluid transport.

We also[Fig alz71844-fig-0004] observed that sparser retinal microvasculature was associated with greater SVD burden. This finding is consistent with our previous OCTA‐based study ^3^ as well as studies in AD and sporadic cerebral SVD demonstrating associations between retinal vascular abnormalities and cerebral microvascular injury.^12^, ^14^, ^34^, ^35^, ^36^ The consistency across different retinal imaging modalities suggests that retinal vascular measures may provide complementary information regarding cerebral vasculature. Nevertheless, because the current CU control group was intentionally selected to have no clinically significant MRI abnormalities, the between‐group differences in SVD burden may have been accentuated. Future studies incorporating control participants with comparable vascular burden but without neurodegenerative disease would be valuable for distinguishing disease‐specific effects from those attributable to cerebrovascular pathology alone.

Sparser retinal microvasculature was additionally associated with poorer global cognitive performance and greater disease severity. We also observed that lower DTI‐ALPS was associated with poorer cognitive performance. Furthermore, exploratory interaction analyses suggested that retinal vascular FD and DTI‐ALPS together were associated with cognitive performance, while retinal microvasculature and SVD burden were jointly associated with disease severity. Because of the modest sample size and cross‐sectional study design, these interaction analyses should be considered hypothesis generating rather than confirmatory. Larger independent cohorts will be required to determine whether these relationships are reproducible.

The exploratory genetic analyses should likewise be interpreted cautiously. Although mutation carriers demonstrated sparser retinal microvasculature than non‐carriers, the number of pathogenic mutation carriers was limited, and different genetic forms of FTD are known to exhibit distinct molecular mechanisms and neuroimaging phenotypes. Consequently, combining mutation carriers into a single group may obscure gene‐specific effects. Larger genetically stratified cohorts will be necessary to determine whether retinal vascular alterations differ among genetic subtypes in FTD.

From a translational perspective, retinal imaging has several practical advantages as a potential biomarker. CFP is inexpensive, rapid, non‐invasive, and widely available across clinical settings. When combined with MRI‐derived markers of vascular and glymphatic‐related abnormalities, retinal microvasculature may contribute to multimodal approaches for characterizing neurovascular changes in FTD. However, whether retinal vascular alterations precede neurodegeneration, parallel disease progression, or simply reflect concurrent vascular injury remains unknown and requires longitudinal investigation.

Several limitations should be acknowledged. First, the study included a relatively small sample size, limiting statistical power, particularly for subgroup, interaction, and genetic analyses. Accordingly, these findings should be regarded as exploratory. Second, although multiple comparisons were addressed using Bonferroni correction, replication in larger independent cohorts remains essential. Third, the cohort was predominantly composed of patients with bvFTD; therefore, the findings should not be generalized to all clinical FTD syndromes. Fourth, FTD diagnoses were based on established clinical criteria without neuropathological confirmation or systematic tau biomarker assessment. Fifth, retinal microvasculature may be influenced by ocular and systemic factors such as refractive error, axial length, and smoking status, which were not comprehensively measured in the present study. Finally, DTI‐ALPS and PVeD are indirect MRI‐derived surrogate markers of glymphatic‐related function rather than direct measures of glymphatic activity, and the cross‐sectional design precludes conclusions regarding causality or temporal relationships among retinal vascular alterations, cerebral vascular injury, fluid transport abnormalities, and cognitive decline. Another limitation in our study is the inclusion of CU controls with no clinically significant MRI abnormalities, which increases the between‐group differences and does not allow separation of disease‐specific effects from vascular pathology alone. Recruiting age‐matched controls with comparable SVD burden but without neurodegenerative disease would indeed represent an informative comparison for future studies.

In conclusion, this study demonstrates that sparser retinal microvasculature is associated with MRI‐derived markers of SVD and glymphatic‐related diffusion abnormalities in a clinically diagnosed, predominantly bvFTD cohort. These findings suggest that retinal vascular architecture may provide complementary information regarding neurovascular abnormalities in FTD and support further investigation of retinal imaging as part of multimodal biomarker strategies.

## AUTHOR CONTRIBUTIONS

*Study concept and design*: William Robert Kwapong, Yufei Chen, Liyong Wu. *Data acquisition*: William Robert Kwapong, Yufei Chen, Xu Chen, Xuxiang Zhang, Liyong Wu, Yuan Gao, Jiahui Hou, Hua Lu, Yuxuan Xu, Qianqian He, Ruoyu Jiao. *Data analysis and interpretation*: William Robert Kwapong, Yufei Chen, Min Chu, Liyong Wu. *Drafting of the manuscript*: William Robert Kwapong, Yufei Chen, Liyong Wu. *Critical review of the manuscript*: William Robert Kwapong, Yufei Chen, Liyong Wu. All authors reviewed and approved this version of the manuscript.

## CONFLICT OF INTEREST STATEMENT

The authors declare that they have no conflicts of interest. The sponsor had no role in the study design, data collection, or writing of the manuscript. Author disclosures are available in the Supporting Information.

## HUMAN ETHICS APPROVAL

The protocol of this study was approved by the ethics committee of Xuanwu Hospital, Capital Medical University (Ethics Number: 2025 011‐001).

## CONSENT STATEMENT

All participants or their legal guardians provided written informed consent before enrolling in the study.

## Supporting information



Supporting Information: alz71844‐sup‐0001‐SuppMat.docx

Supporting information: alz71844‐sup‐0002‐ICMJE.pdf

## Data Availability

The dataset generated and analyzed for this study will be made available by the corresponding author upon reasonable request.
