# Supplementary material for: Retinal microvascular changes are related to indicators of glymphatic dysfunction and small vessel disease in frontotemporal dementia
Source: Alzheimers Dement. 2026 Sep 17;22(9):e71844. doi: 10.1002/alz.71844 (PMC13586002; doi:10.1002/alz.71844)
Supplement: Supplementary file 2 [file ALZ-22-e71844-s001.docx]

**Supplementary Table 1. The association between eye-specific retinal microvasculature, neuroimaging measures and in FTD.** The same confounding factors as in the primary analysis were adjusted.

| **Variables** | **DTI-ALPS** | | **PVeD** | | **SVD burden** | |
| --- | --- | --- | --- | --- | --- | --- |
|  | β (95% CIs) | *p* value | β (95% CIs) | *p* value | β (95% CIs) | *p* value |
| FD (OD) | 0.045 (0.005 – 0.086) | **0.050** | 0.040 (-0.003 – 0.083) | 0.097 | -0.017 (-0.049 – 0.014) | 0.282 |
| FD (OS) | 0.053 (-0.0003 – 0.106) | 0.077 | 0.036 (-0.023 – 0.095) | 0.262 | -0.050 (-0.082 - -0.017) | **0.006** |
| Arterioles (OD) | 0.047 (0.015 – 0.081) | **0.016** | 0.028 (-0.013 – 0.069) | 0.203 | -0.023 (-0.051 – 0.004) | 0.104 |
| Arterioles (OS) | 0.066 (0.005 – 0.126) | 0.057 | 0.034 (-0.035 – 0.104) | 0.355 | -0.051 (-0.086 - -0.015) | **0.010** |
| Venules (OD) | 0.040 (0.013 – 0.067) | **0.014** | 0.041 (0.015 – 0.068) | **0.011** | -0.010 (-0.033 – 0.014) | 0.425 |
| Venules (OS) | 0.054 (0.007 – 0.101) | **0.048** | 0.044 (-0.007 – 0.096) | 0.122 | -0.045 (-0.074 - -0.016) | **0.006** |
